# Improving Performance of an Open Cell Aluminium Foam through Electro-Deposition of Nickel

**DOI:** 10.3390/ma12010133

**Published:** 2019-01-03

**Authors:** Silvio Genna, Federica Trovalusci, Nadia Ucciardello, Vincenzo Tagliaferri

**Affiliations:** Department of Enterprise Engineering, University of Rome Tor Vergata, Via del Politecnico, 1, 00133 Rome, Italy; federica.trovalusci@uniroma2.it (F.T.); nadia.ucciardello@uniroma2.it (N.U.); taglia@uniroma2.it (V.T.)

**Keywords:** aluminium foams, electro-deposition, annealing, compression test, corrosion test

## Abstract

The aim of this work is to investigate the mechanical performances and corrosion resistance of open-cell aluminium foams with an electroplated nickel coating. The influence of two different electrolytic solutions on mechanical properties and corrosion resistance was studied: The Watts solution (nickel sulphate-based solution) and a nickel sulphamate solution (widely adopted). Scanning electron microscopy and stereoscopic analysis allowed for the estimation of the coating uniformity and adhesion to the substrate. In order to assess the improvement of performances, compression and corrosion tests were performed on coated and uncoated foams. In addition, annealing was investigated in relation to different operational parameters, related both to electro-deposition (electrolyte, deposition current and time) and to annealing (treatment temperature). From the results, the yield stress and the corrosion resistance improved. Moreover, the annealing at increasing temperature was found to reduce the yield stress, but Ni-coated foams showed higher values of stress for all the considered treatment temperatures.

## 1. Introduction

Metal foams are porous materials characterized by a complex 3D structure, which confers to them interesting properties in terms of light weight, mechanical, thermal and acoustic performances. In particular, metal foams are widely adopted because of their higher specific strength and stiffness, as compared to monolithic equivalents. The characteristics of these porous materials make them suitable for several industrial applications [1,2], from energy-absorbing systems [3] and fuel cells [4], to cooling systems and heat exchangers [5,6,7]. In the latter case, open cell structures are used because of their very high thermal conductivity. From numerical analysis, heat exchangers, based on metal foams, are superior as compared to conventional finned exchangers at no excess cost [8]. Moreover, by depositing appropriate coatings on the foam, it is possible to improve some properties, such as heat exchange and energy absorption capacity. To produce more compact heat exchangers, heat exchange capacity can be improved by using copper coated foams [9]. At the same time, this seems to be a promising solution to increase absorption capacity. By comparing Cu-coated and uncoated aluminium foams with the same overall thickness (i.e., same effective volume), the coated ones had double energy absorption capacity [10]. Recently, electro deposition of copper on aluminium open cell foams was studied, with the purpose to improve their thermal properties; moreover, an analytical model was proposed to estimate the characteristics of the coating [11]. By applying this model, it was possible to identify the optimal operational parameters to be used in electro-deposition. 

The interest in copper deposition is also due to the fact that coppering can be used as the base for further deposition of other materials, such as nickel [12].

Recent studies showed that the electro-deposition of nickel determined an improvement in mechanical properties of open cell aluminium foams [13,14]. Generally, metallic foams are mechanically characterized by performing a uniaxial compression test [15,16,17], while in [13] an innovative non-contact characterization procedure was defined to evaluate the mechanical performance of the coated foams, with an increased strain measurement resolution.

In the scientific literature, few studies focus on nickel coated foams, the complexity of covering the inner zone of cellular structures with this material [13] and their final properties [18,19,20]. However, they showed that the increase in density was related to the increase in mechanical properties. Other studies [21,22] introduced an analysis that consider the relative properties of the deposited material, and the beam-bending mechanics of how this influences the overall material behaviour.

Models were also developed to estimate the improvement in mechanical properties due to the metallic coating [23,24]. They can be adopted to study the influence of the parameters on the mechanical properties.

The aim of this work is to study the influence of two different electrolytic solutions on the mechanical properties and corrosion resistance of an aluminium foam electro-deposited with nickel: The Watts solution (i.e., a nickel sulphate-based solution, as adopted in [13]) and a nickel sulphamate solution (widely adopted) [25]. For both deposition types, the influence of different electro-deposition parameters, such as electrolyte, direct current and deposition time were changed and tested. The coating uniformity and adhesion to the substrate were assessed by scanning electron microscopy and stereoscopic analysis. In order to assess the improvement of performances, compression and corrosion tests were performed on coated and uncoated foams. In addition, annealing was investigated in relation to different operational parameters (electrolyte, deposition current and time), as high temperature determines a microstructural variation of aluminium, as observed in [26].

## 2. Materials and Methods

### 2.1. Coatings Preparation

Parallelepiped samples, 20 × 20 mm^2^ in size and 30 mm in height, were obtained from the same commercial panel of open cell foam (Duocel^®^ by ERG Material, Oakland, CA, USA). It was made of 6101 T6 aluminium alloy and the number of pores per inches (PPI) was 10. This foam is fabricated by the replication of polymeric pattern [1].

Aluminium foam substrates were coated via electro-deposition. An electrolytic cell at 25 °C was used. Two different electrolytic solutions were studied: the Watts solution (i.e., a nickel sulphate-based solution) (NiSO_4_·6H_2_O: 225 g/L; NiCl_2_·6H_2_O: 30 g/L; H_3_BO_3_: 30 g/L), as adopted in [13], and a nickel sulphamate solution (Ni(NH_2_SO_3_)_2_·4H_2_O: 300 g/L; NiCl_2_·6H_2_O: 30 g/L; H_3_BO_3_: 30 g/L), generally adopted for the deposition of functional coatings or for electroforming [25]. 

The main issue with electro deposition of foams is with the penetration of the Ni ions into the cellular structure, which is a very complex 3D structure, and; therefore, the obtaining of a very uniform thickness of Ni on all the brackets of foams. In order to overcome this issue, a magnetic agitator, working at 3 rpm, was used to keep in agitation the bath during the deposition. The consumable anode was composed by four Ni plates, 40 × 40 × 3 mm^3^ in size, forming a hollow parallelepiped, which surrounded the sample. The cathode was the foam and was connected to the current generator and immersed in the electrolyte. Different values of operational parameters were considered: direct current (DC) was set at 1, 1.5 and 2 A; deposition time ranged from 10 to 120 min. Such current values were chosen on the basis of previous works [11,13] and on the basis of pre-tests, showing that the further increase of current determined a lack of continuity of the coating.

### 2.2. Experimental Procedure

A characterization procedure was defined in this work with the aim to identify the effect of the parameters, adopted during deposition or annealing, on the resulting foams. In particular, the thickness and the adhesion of the coating were evaluated by scanning electron microscopy (FEG-SEM Leo Supra 35, Zeiss, Oberkochen, Germany) and stereoscopic microscope (SMZ745T, Nikon, Düsseldorf, Germany). Both the external face and the interior of the foam were analysed.

In the mechanical characterization tests, the compression of the foams was performed by a universal testing machine (Alliance RT 50, MTS, Berlin, Germany); the crosshead speed was 10 mm/min and the three zones of the stress-strain curves (initial elastic zone, plateau zone and failure zone) were analysed [23]. Three replications were performed for each condition.

The effect of annealing on mechanical properties was also investigated. Annealing for 30 min at various temperatures, from 150 °C up to 450 °C, was performed in a convection oven, both on coated and uncoated foams for comparison. 

The comparison was in terms of yield stress, evaluated by performing compression tests in the aforementioned conditions. Microstructural analysis of samples, after annealing tests, was performed to verify the possible occurrence of defects.

**Figure 1:**
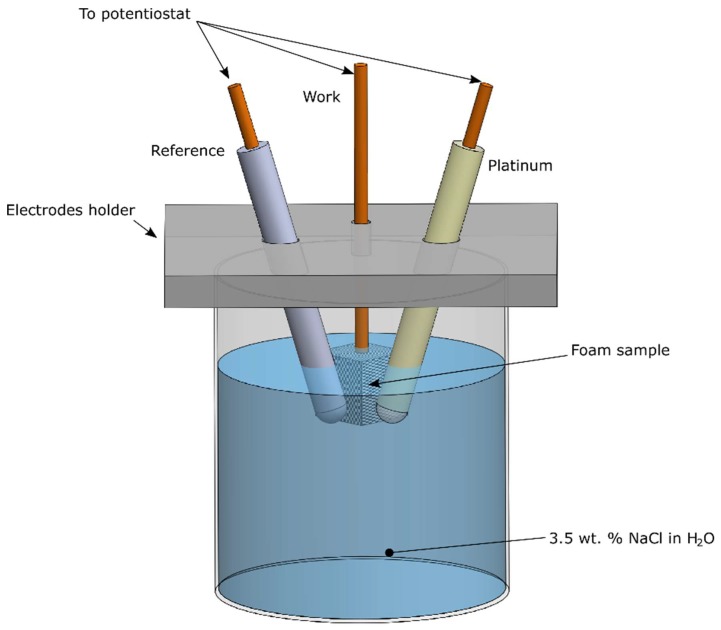
Schematic of the corrosion test.

In addition, corrosion tests were performed on the samples to evaluate the protection offered by the Ni coating on the aluminium foam. In order to study the corrosion behaviour, the “VoltaLab” potentiostat (VoltaLab 80, Radiometer Analytical, Villeurbanne, France) was adopted to produce the Tafel curves of the annealed samples. The auxiliary electrode, used for the test, was made in platinum, whereas the reference one was a saturated calomel electrode (SCE), according to the schematic of Figure 1. The corrosion resistance was evaluated in water solution with 3.5 wt. % of NaCl; before the tests, the solution was degassed.

## 3. Results and Discussion

The amount of deposited nickel (*P*%), calculated as the variation of the sample mass before and after electro-deposition, was chosen as the principal parameter for evaluating the standard of the deposition process. It is a mass ratio and can be expressed as a function of the molar mass of nickel ($MM_{Ni}$) and the number of moles of deposited nickel ($n_{Ni}$):

(1) $$P\%=\frac{MM_{Ni}\cdot n_{Ni}}{\rho \cdot V}\;100$$

The reduction half-reaction that occurs at the cathode:

(2) $$Ni^{2+}+\;2e^{-}\to \;Ni^{0}$$

suggests that, for every mole of Ni ion that is deposited, two moles of electrons are required; therefore, *P*% can be written as:

(3) $$P\%=\frac{MM_{Ni}\cdot n_{e^{-}}}{\rho \cdot V\cdot 2}\cdot 100$$

Being *e* the electric charge of electron, *t* the time, *i* the electric current and *N_a_* the Avogadro’s number, it is possible to change the previous equation, in agreement with Antenucci [11]:

(4) $$P\%=\;\frac{MM_{Ni}}{N_{a}\cdot 2\cdot e}\cdot \;\frac{i\cdot t}{\rho \cdot V}\;\cdot 100$$

obtaining a rearrangement of the Faraday law, in which the first fraction is a constant and assumes the value 1.83 × 10^−3^ A min/kg.

Equation (4) shows the relationship that exists between *P*% and the operational parameters of the electro-deposition process. In particular, *P*% is directly proportional to current and time and inversely proportional to sample mass. Attention must be paid to the value assumed by *ρ*, that is not constant and has to be calculated for each sample. Thus, each sample was weighted before and after electro-deposition, the volume was estimated and then the density was calculated.

**Figure 2:**
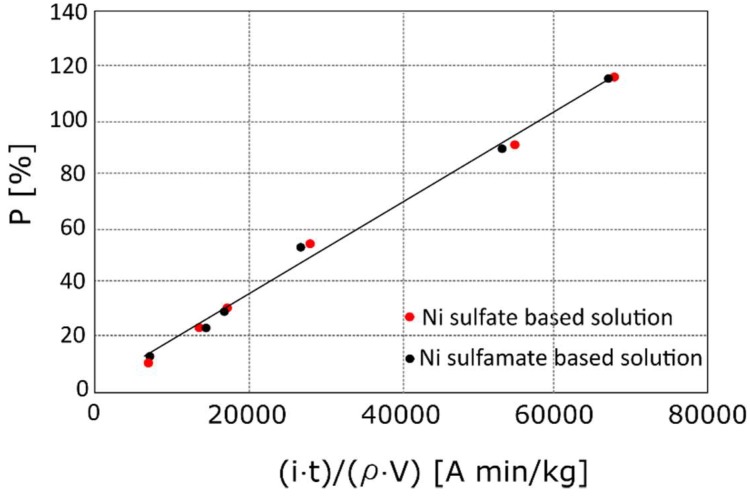
Percentages of nickel deposited *P*% as a function of the variable term of Equation (4).

In Figure 2, the percentages of nickel deposited *P*% as a function of the variable term of Equation (4) is reported; the slope of the linear trend (the first fraction of Equation (4)) assumed the value 1.8 × 10^−3^ A min/kg regardless of the electrolyte, which was in good agreement to the calculated value. Thus, Equation (4) accurately predicts the amount of deposited Ni. It is worth noting that the equation can be adopted in the case of an open cell foam, for both Ni sulphate and Ni sulphamate solutions.

Table 1 summarizes the values of density and the amount of nickel deposited (calculated by Equation (4)) achieved after electro-deposition at 1.5 A (intermediate level), according to the process time; the initial density (i.e., the uncoated foam density) was 180 ± 20 kg/m^3^. Significant differences between the two adopted solutions (Ni sulphate/sulphamate) were not observed. The density increase was about 20 kg/m^3^ for the lower deposition time (10 min), and about 230 kg/m^3^ for the higher time (120 min). 

The density of the foam is strictly related to its mechanical properties, the more the foam is dense the more it is resistant [27]. 

The uniformity and the thickness of the electroplated coating were assessed by SEM and stereoscopic analysis. 

**Figure 3:**
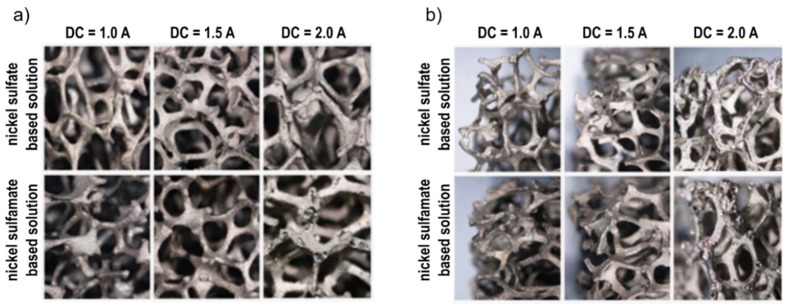
Images of foams after Ni electro-deposition for 60 min and different DC (1.0, 1.5 and 2 A): (**a**) internal part; (**b**) external part.

Figure 3 shows the stereoscopic images of samples obtained at the intermediate deposition time (60 min) and different DCs (1.0, 1.5, 2.0 A), for both the solutions. From the figure, the uniformity of the covering layer of nickel on the substrate was verified for both the faces, external and interior. However, this observation is particularly useful for choosing the optimum value of direct current to be adopted for Ni electro-deposition: for all the values of time, by increasing the intensity of the current from 1 to 1.5 A, both the external face and the interior of the foam were covered in a better way, in fact, lower DC did not generate forces strong enough to guarantee a correct deposition, as found in [11,13]. On the other hand, a further increase (up to 2 A) determined a lack of continuity of the coating, as clearly visible in Figure 3, where small points were visible on the last pictures. This phenomenon can be contributed to the formation of hydrogen bubbles over the external surface of the foam. 

The same considerations can be drawn regardless of the electrolyte. For this reason, the focus was mainly on the electro-deposition at 1.5 A, corresponding to the higher final density, with no defects; the resulting samples were considered for the subsequent mechanical and corrosion tests. 

**Figure 4:**
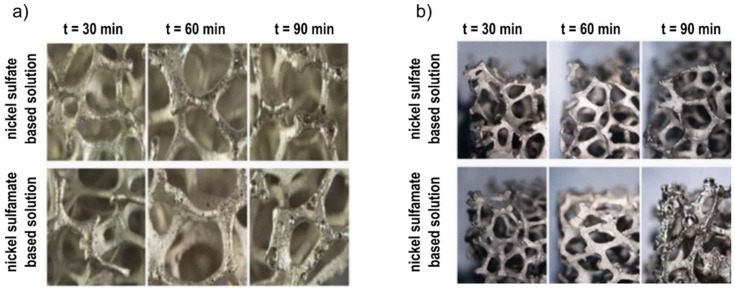
Images of foams after Ni electro-deposition at 1.5 A and different deposition times: (**a**) internal part; (**b**) external part.

In Figure 4, the stereoscopic images of samples obtained at 1.5 A and different deposition times (30, 60, 90 min) are reported, for both the solutions. Similar to the effect of DC variation, a damaged coating was observable at the highest process time. Moreover, coatings obtained by using the sulphamate solution showed a greater predisposition to the formation of surface defects, and which were amplified by increasing the deposition time. From the above, the best electro-deposition condition can be obtained at 1.5 A for 60 min, with the sulphate solution.

**Figure 5:**
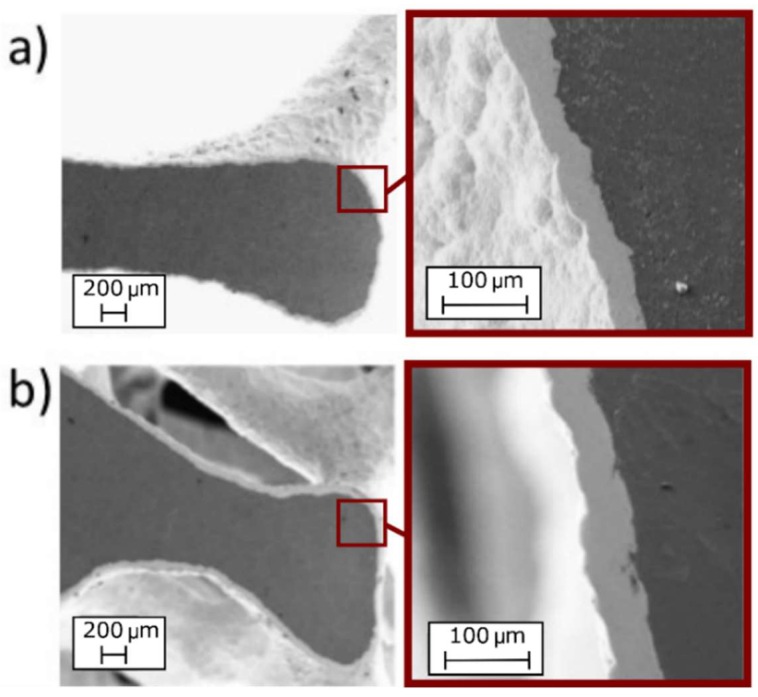
SEM images of foams after Ni electro-deposition at 1.5 A and 60 min; adhesion and thickness of the coating along the section: (**a**) nickel sulphate-based solution; (**b**) nickel sulphamate-based solution.

In order to assess if the solution could have an influence on the adhesion between the coating and the substrate, SEM analysis of the foams (electroplated at 1.5 A and 60 min) were performed. In Figure 5 the images obtained by SEM are reported. From the images, the uniformity and good adhesion of the coatings along the section are clearly visible, for both the solutions. 

**Figure 6:**
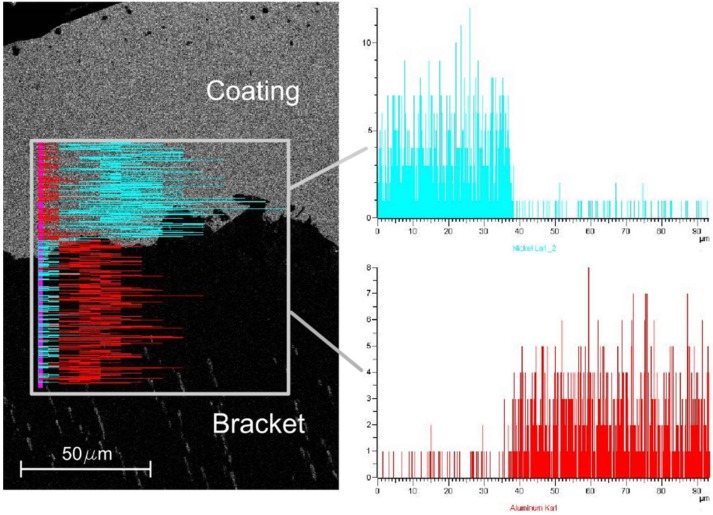
EDS analysis of the foam after Ni electro-deposition at 1.5 A and 60 min with the nickel sulphate-based solution.

In addition, the Energy Dispersive Spectrometer analysis (EDS Inca 300, Oxford Instruments Ltd., Abingdon, UK) of the foam after Ni electro-deposition at 1.5 A and 60 min with the nickel sulphate-based solution was performed, as reported in Figure 6.

**Figure 7:**
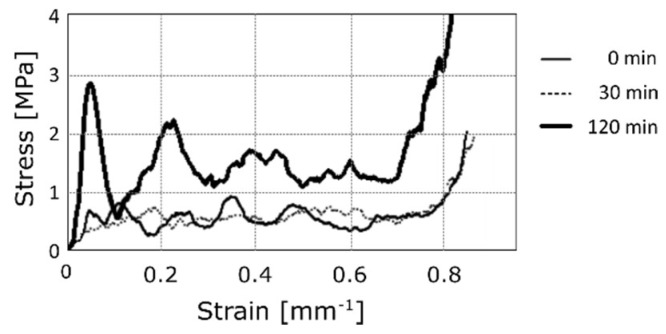
Compression test: Typical stress-strain curves for different deposition times at 1.5 A, in the nickel sulphate-based solution.

In order to characterize the mechanical performance of the coated foams, compression tests were performed. Figure 7 shows the typical stress-strain curves for different deposition times at 1.5 A for the nickel sulphate solution. It is worth noting that the irregularities correspond to the progressive initiation of plastic buckling of the brackets in the foam [28,29,30]. According to the scientific literature, three different zones can be identified: first, the elastic zone, then a plateau, and last, the failure of the material. After the elastic zone, most of the samples show a drop in resistance; this trend has already been verified in other works [18]. In the figure, the mechanical behaviour of the uncoated aluminium foam (deposition time of 0 min) is compared to the performances exhibited by the coated ones, at increasing deposition times, using the nickel sulphate solution, which seemed to minimize the formation of surface defects. A sliding up of curves proportional to the deposition time, and thus to the density, can be observed. Stiffness seemed to be unaffected by the electro-deposition of nickel. On the contrary, the yield stress was clearly improved. 

**Figure 8:**
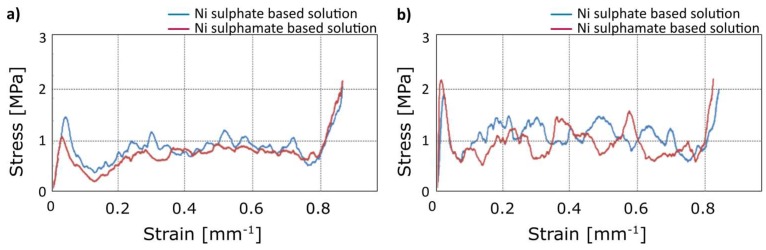
Compression test: Comparison of stress-strain curves for different electrolytes at 1.5 A: (**a**) 60 min; (**b**) 90 min.

Significant differences in terms of mechanical behaviour under compression load were not observed using different electrolytes, as suggested by the comparison of stress-strain curves in Figure 8. This was expected, as the good adhesion between the coating and the substrate was verified by the SEM analysis for both the solutions.

From the above, the coatings obtained by using the sulphamate solution showed a greater predisposition to formation of surface defects, at the same time this electrolyte conferred to the foam mechanical properties similar to the ones obtainable by sulphate solution. Thus, further considerations were done referring to the electro-deposition by the sulphate solution. 

**Figure 9:**
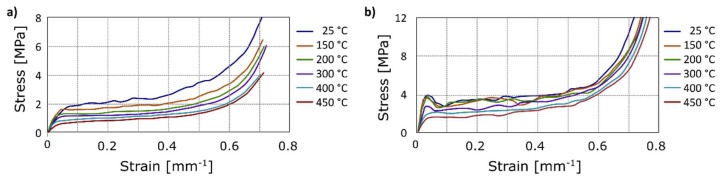
Compression test: Stress-strain curves after annealing at different temperature, (**a**) uncoated foams; (**b**) Ni-coated foams (nickel sulphate solution).

**Figure 10:**
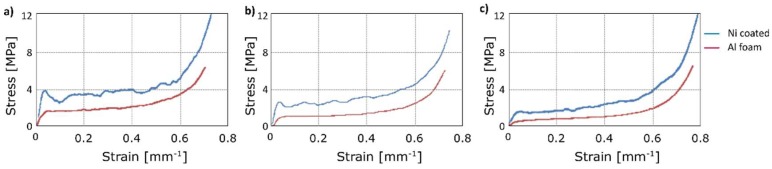
Compression test: Stress-strain curves at (**a**) 25 °C; after annealing at (**b**) 300 °C; (**c**) 450 °C.

In the following, the compression tests after annealing are reported. In Figure 9, the mechanical behaviour of aluminium foam and Ni-coated aluminium foams is reported at increasing temperatures of annealing. The initial density was the same for all the compared samples. As expected, the yield stress was reduced after annealing at increasing temperature. Figure 10 allows a clear comparison, it suggests that the yield stress of the aluminium foam at room temperature was close to 1.8 MPa, this value decreased with increasing temperatures of annealing up to 0.7 MPa (at 450 °C). This was due to long treatment time or high treatment temperatures, which determined a microstructural variation of aluminium, in particular the increase of the grain size, as known in the scientific literature [31].

The results are summarized in Table 2: Ni-coated foams showed higher values of stress for all the considered temperatures of annealing. This fundamental aspect means that Ni-coated foams could also be used after exposure to elevated temperature, whereas aluminium foams do not provide adequate mechanical properties. 

**Figure 11:**
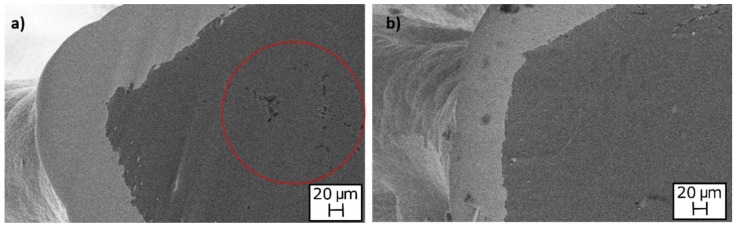
SEM images of Ni-coated foams after annealing at: (**a**) 300 °C; (**b**) 150 °C.

In addition, SEM analysis was performed to establish the maximum temperature that did not cause defects. It was found that the annealing treatment at 300 °C determined porosity inside the aluminium (red circle in Figure 11a), which could reduce the mechanical strength of the aluminium foam. This degradation did not occur at temperatures lower than 300 °C (Figure 11b). These observations agree with the results of mechanical characterization, which showed significant reduction of mechanical performances after annealing performed at temperatures higher than 300 °C. However, as visible in Figure 10a, the coating was still adherent to the substrate at a high temperature. 

Based on the discussed results, the improvement of properties achieved by the electro-deposition of nickel was assessed. In fact, regardless of annealing conditions, Ni-coated foams showed higher performance than aluminium foams also after exposure to high temperature, up to 450 °C. 

**Figure 12:**
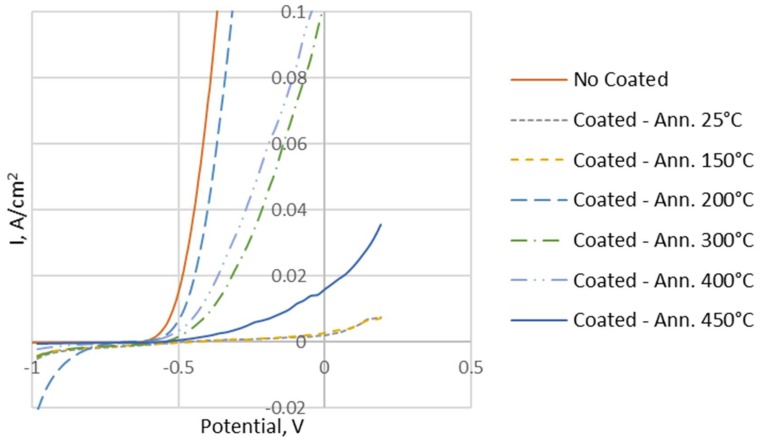
Tafel curves of uncoated and coated samples.

In addition, corrosion tests were performed on the samples to evaluate the protection offered by the Ni coating on the aluminium foam at increasing annealing temperatures. The results are shown in Table 3 and Figure 12. 

From the analysis of Figure 12, the improvement on corrosion resistance, of the coated samples as compared to the uncoated sample, is clearly visible. 

From the above, the Ni coating improved the mechanical and corrosion performance of an aluminium open cell, even at high temperatures, allowing for their adoption, whereas uncoated aluminium foams did not provide an adequate mechanical (and corrosion) performance.

## 4. Conclusions

The present work investigated the mechanical performances and corrosion resistance of open-cell aluminium foams with an electroplated nickel coating. Two different electrolytic solutions were indagated to obtain the coatings: The Watts solution (nickel sulphate-based solution) and a nickel sulphamate solution (widely adopted). Scanning electron microscopy and stereoscopic analysis was used to estimate the coating uniformity and adhesion to the substrate. The evaluation of properties of the resulting foams was based on compression and corrosion tests. Based on the experimental results, within the checked values, the following conclusions can be drawn:

(1) Both the external face and the interior of the foams were correctly coated by adopting a DC of 1.5 A, which prevented the formation of hydrogen bubbles over the external surface of the foams;
(2) The sulphamate solution (i.e., the widely adopted one) showed a higher predisposition to the formation of hydrogen gas and; therefore, of surface defects on the foam;
(3) The optimal duration of deposition was 60 min; which allowed for a homogenous and satisfactory covering of samples to be obtained with the considered shape and dimensions;
(4) The amounts of nickel deposited on the specimens was almost the same for the two solutions;
(5) Mechanical properties of coated foams were similar, regardless of the electrolyte;
(6) The coating improved the yield, whereas the stiffness was found to be unaffected by the deposition of Ni;
(7) The yield stress was reduced after annealing at increasing temperature, but Ni-coated foams showed higher values of stress for all the considered temperatures;
(8) The Ni coating allowed a higher corrosion current, even at high temperatures.

## Figures and Tables

**Table 1:** The coated foam density and amount of nickel deposited at 1.5 A.

Deposition Time (min)	Nickel Sulphate		Nickel Sulphamate	
	Final Density (kg/m^3^)	*P*%	Final Density (kg/m^3^)	*P*%
10	199 ± 10	10.88	201 ± 8	11.47
20	205 ± 15	23.43	200 ± 19	23.72
30	258 ± 18	30.59	256 ± 11	30.24
60	292 ± 10	57.71	261 ± 21	54.44
90	356 ± 12	92.23	358 ± 14	90.72
120	422 ± 17	116.84	420 ± 28	117.49

**Table 2:** Compression test: Maximum stress in the range 0–0.1 mm^−1^ strain and plateau length after annealing.

Annealing Temperature (°C)	Plateau Length (mm)		Maximum Stress (MPa)	
	Aluminium Foam	Ni-Coated Foam	Aluminium Foam	Ni-Coated Foam
25	0.26	0.37	1.72	3.88
150	0.31	0.39	1.61	3.72
200	0.35	0.41	1.21	3.75
300	0.35	0.38	1.16	2.87
400	0.38	0.39	0.89	2.15
450	0.37	0.40	0.69	1.63

**Table 3:** Corrosion test: Tafel current of uncoated and coated samples.

Annealing Temperature (°C)	I (mA/cm^2^)	Sample
*-*	0.101 ± 0.04	uncoated
25	0.278 ± 0.07	coated
150	0.238 ± 0.09	
200	0.665 ± 0.08	
300	0.852 ± 0.05	
400	0.683 ± 0.07	
450	0.149 ± 0.09	
